# Protein kinase C activation mediates interferon-β-induced neuronal excitability changes in neocortical pyramidal neurons

**DOI:** 10.1186/s12974-014-0185-4

**Published:** 2014-10-29

**Authors:** Olivia Reetz, Konstantin Stadler, Ulf Strauss

**Affiliations:** Institute of Cell Biology & Neurobiology, Charité – Universitätsmedizin Berlin, Charitéplatz 1, 10117 Berlin, Germany; Industrial Ecology Programme, NTNU - Norwegian University of Science and Technology, Trondheim, Norway

**Keywords:** PKC, IFN, Neuronal inflammation, Nervous - Immune system interactions, Excitability modulation, Layer 5, NEURON

## Abstract

**Background:**

Cytokines are key players in the interactions of the immune and nervous systems. Recently, we showed that such interplay is mediated by type I interferons (IFNs), which elevate the excitability of neocortical pyramidal neurons. A line of indirect evidence suggested that modulation of multiple ion channels underlies the effect. However, which currents are principally involved and how the IFN signaling cascade is linked to the respective ion channels remains elusive.

**Methods:**

We tested several single and combined ionic current modulations using an *in silico* model of a neocortical layer 5 neuron. Subsequently we investigated resulting predictions by whole-cell patch-clamp recordings in layer 5 neurons of *ex vivo* neocortical rat brain slices pharmacologically reproducing or prohibiting neuronal IFN effects.

**Results:**

The amount and type of modulation necessary to replicate IFN effects *in silico* suggested protein kinase C (PKC) activation as link between the type I IFN signaling and ion channel modulations. In line with this, PKC activation with 4β-phorbol 12-myristate 13-acetate (4β-PMA) or Bryostatin1 augmented the excitability of neocortical layer 5 neurons comparable to IFN-β in our *ex vivo* recordings. In detail, both PKC activators attenuated the rheobase and increased the input-output gain as well as the input resistance, thereby augmenting the neuronal excitability. Similar to IFN-β they also left the threshold of action potential generation unaffected. In further support of PKC mediating type I IFN effects, IFN-β, 4β-PMA and Bryostatin1 reduced the amplitude of post-train after-hyperpolarizations in a similar manner. In conjunction with this finding, IFN-β reduced M-currents, which contribute to after-hyperpolarizations and are modulated by PKC. Finally, blocking PKC activation with GF109203X at the catalytic site or calphostin C at the regulatory site prevented the main excitatory effects of IFN-β.

**Conclusion:**

Multiple ion channel modulations underlie the neuromodulatory effect of type I IFNs. PKC activation is both sufficient and necessary for mediating the effect, and links the IFN signaling cascade to the intrinsic ion channels. Therefore, we regard PKC activation as unitary mechanism for the neuromodulatory potential of type I IFNs in neocortical neurons.

**Electronic supplementary material:**

The online version of this article (doi:10.1186/s12974-014-0185-4) contains supplementary material, which is available to authorized users.

## Introduction

IFNs are cytokines with diverse biological capabilities, ranging from their antiviral and anti-inflammatory properties to their regulation of normal and malignant cell growth [1-3]. This accounts for the clinical use of IFNs for various diseases, such as multiple sclerosis or hepatitis C. Among the three known receptor-specific IFN subtypes, type I IFNs (IFN-α and IFN-β) are prominent as the first antiviral reaction to infection. They are produced within the central nervous system by glial cells, mostly microglia and astrocytes, but also by neurons [4]. In addition, type I IFNs directly influence neuronal function. Type I IFN release on the central nervous system, more specifically on the cerebral cortex as for instance in viral encephalitis, affects the excitability level of pyramidal neurons. These effects were presumably due to slight modulation of various ion channels [5]. Such small modulations would cause a more effective (and stable) change of neuronal states as opposed to a large modulation at a single channel [6]. Yet, the precise mechanism of type I IFN neuronal action has remained elusive.

Binding of type I IFNs to their receptors activate receptor-associated Tyk-2 and Jak-1 kinases, resulting in activation of different downstream pathways [7,8]. Common downstream pathways include phosphorylation of the Signal Transducer and Activator of Transcription1 (STAT1) [9], activation of the p38 mitogen-activated protein kinase signaling pathway [3] and activation of the PI3-K pathway [10]. One ubiquitous component of the downstream type I IFN signaling cascade is the activation of various types of protein kinase C (PKC) [2,3]. Activation of PKC isoforms also modulates several voltage sensitive ion channels [11]. In detail, PKC activation reduces the M-type potassium current (*I*_M_) amplitude [12], modulates the hyperpolarization-activated cyclic nucleotide-gated current (*I*_h_) in various expression systems [13,14], changes the opening probability of channels mediating the large-conductance calcium-dependent potassium currents (*I*_BK_) [15] and shifts the activation curve of the persistent sodium currents (*I*_Nap_) [16].

To explore whether PKC activation and consecutive ion current changes mediate neuronal type I IFN effects, we here used a combined approach of *in silico* and *ex vivo* methods. We focused on pyramidal neocortical layer 5 neurons because they are well characterized in terms of content and distribution of ionic currents, expression of dendritic IFN-β receptors and response to type I IFNs [5] under neuroinflammatory conditions [17].

This study corroborates that neuromodulatory effects of type I IFNs are based on multiple modulations of intrinsic ion channels. Combining the results of exploratory analysis by *in silico* modeling with those from a number of comprehensive *ex vivo* experiments we present PKC activation as unitary mechanism linking the IFN signaling cascade to these ion channels.

## Methods

### Interferon and PKC activators/inhibitors

Chinese hamster ovary-derived recombinant rat IFN-β protein (U-CyTech, Utrecht, Netherlands) was dissolved in sterile double-distilled water to a concentration of 10^5^ IU and stored at –20°C. The final concentration was 1,000 IU IFN-β ml^-1^, as this was previously shown to effectively increase suprathreshold responses [5] and is assumed to occur during viral infections [17,18]. PKC activators 4β-phorbol 12-myristate 13-acetate (4β-PMA) or Bryostatin1 and PKC inhibitors GF109203X (also known as BisI or Gö6850) or calphostin C (all Tocris Bioscience, Bristol, UK) were dissolved in 99.8% Dimethyl sufoxide (Sigma-Aldrich, Steinheim, Germany) to stock concentrations of 10 mM (4β-PMA, Bryostatin1, GF109203X) or 1 mM (calphostin C) and stored at –20°C. The final concentrations in artificial cerebrospinal fluid (ACSF, for content see patch clamp recordings) were 1 μM (4β-PMA, Bryostatin1, GF109203X, for all: 10 μl to 100 ml ACSF) and 100 nM (calphostin C, 10 μl to 100 ml ACSF). In all cases the final Dimethyl sulfoxide percentage accounted for 0.01%.

### Animals and slice preparation

Juvenile male Wistar rats between postnatal day (P)11 and P27 (Research Institutes for experimental medicine (FEM), Berlin, Germany) were used throughout the study. Animals were kept under standard laboratory conditions and all treatments were performed in agreement with the European Communities Council Directive of 22 September 2010 (2010/63/EU). Animals were deeply anesthetized with isoflurane (Abbott GmbH, Wiesbaden, Germany) and decapitated. The brain was quickly removed and immediately transferred to cold (2 to 5°C) sucrose artificial cerebrospinal fluid (sACSF) containing (in mM): 85 NaCl (Sigma-Aldrich), 2.5 KCl, 1 NaH_2_PO_4_, 7 MgCl_2_, 26 NaHCO_3_, 10 D(+)-glucose, 50 sucrose and 0.5 CaCl_2_ (all from Merck, Darmstadt, Germany) bubbled with a gas mixture of 95% O_2_ and 5% CO_2_. Using a vibrating microtome (VT1200, Leica, Nussloch, Germany), cortical brain slices of 300 to 400 μm containing the somatosensory cortex were cut in 2 to 5°C cold sACSF. Slices were transferred to 33 ± 1°C warm sACSF to recover for at least 0.5 hours and kept in sACSF at room temperature.

### Patch clamp recordings

Brain slices were transferred to a recording chamber constantly perfused with 32 to 34°C warm ACSF containing (in mM): 119 NaCl (Sigma-Aldrich), 2.5 KCl, 1 NaH_2_PO_4_, 1.3 MgCl_2_, 26 NaHCO_3_, 10 D (+)-glucose and 2.5 CaCl_2_ (all from Merck, Darmstadt, Germany) bubbled with a gas mixture of 95% O_2_ and 5% CO_2_. Cortical pyramidal neurons were visualized in layer 5 with an Axioskop 2 FS + microscope (Carl Zeiss MicroImaging GmbH, Göttingen, Germany) equipped with infrared differential interference contrast. Patch pipettes were pulled (P-97 micropipette puller, Sutter Instruments, Novato, CA, USA) to a resistance of 3 to 5 MΩ. For recordings with 4β-PMA and Bryostatin1 intracellular solution comprised (in mM) 120 K-gluconate, 10 Na-phosphocreatine, 11 EGTA, 2 Mg^2+^ATP, 0.3 Tris-GTP (Sigma-Aldrich), 10 KCl, 1 MgCl_2_, 1 CaCl_2_ and 10 HEPES. For experiments with IFN-β, pipette solution contained (in mM): 120 K-methylsulphate (KMeSO_4_) (ICN Biomedical Inc, California, USA), 20 KCl, 14 Na-phosphocreatine, 4 NaCl, 0.5 EGTA, 10 HEPES, 4 Mg^2+^-ATP, 0.3 Tris-GTP and 0.1 cAMP (Sigma-Aldrich). The pH of intracellular solutions was adjusted with KOH (Carl Roth, Karlsruhe, Germany) to 7.2.

### Data and statistical analysis

Experiments were recorded with an EPC-10 amplifier (HEKA, Lambrecht, Germany) and controlled by PatchMaster v2.32 software (HEKA). Data were filtered with 3 or 10 kHz and sampled with 10 or 20 kHz, respectively. Offline analysis was performed with FitMaster (HEKA) and Origin 8.5 (Origin Labs, Northampton, MA, USA).

Input resistance was calculated from a linear fit of the I-V plot (data points of the current pulse of ±50 pA and 0 pA). The relationship between input current and number of action potential (F-I) is characterized by two parameters: rheobase and F-I slope. Both were calculated by a linear fit of the F-I plot in its first, assumingly physiologically most relevant, linear proportion. The rheobase equates intersection of fit and abscissa. Data are given as mean ± SEM throughout. Statistical analyses were performed with paired *t*-tests. In case of non-normal distribution or for datasets smaller than n = 8 nonparametric tests for paired data (Wilcoxon signed rank tests) were used. Results were regarded as statistically significant when *P* < 0.05.

### Layer 5 pyramidal neuron model

**Figure 1:**
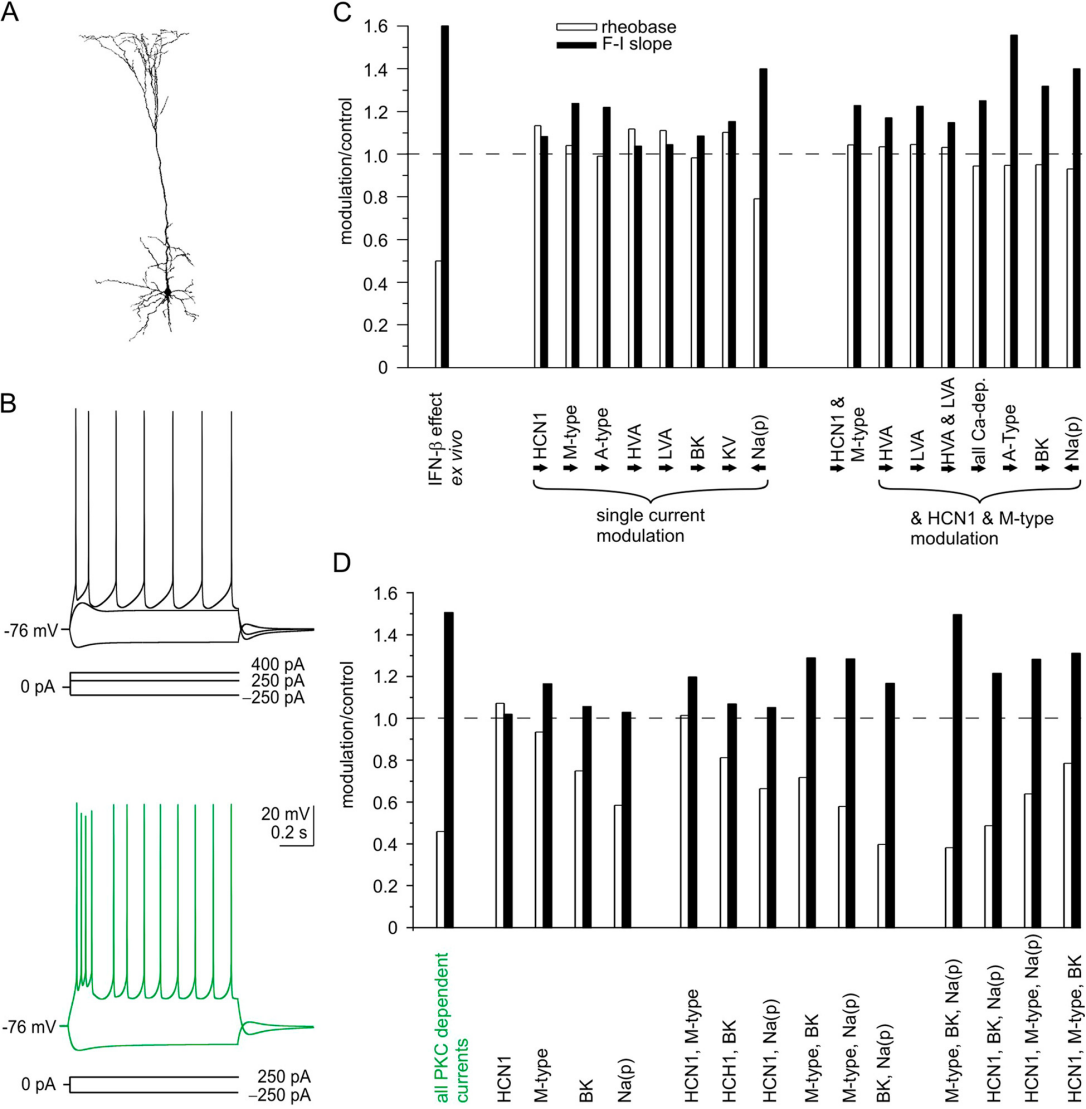
**Concerted protein kinase C modulation of ionic conductances mimic type I IFN effects on suprathreshold excitability** ***in silico*** **. (A)** Cell geometry used in the model. **(B)** Voltage response of the simulated neuron to different input currents for control conditions (black traces) and after simulating protein kinase C (PKC)-mediated ionic current modulations (green traces). Virtual 1-second rectangular current injections are given below trace families. Note that the virtual current injection of 250 pA did not elicit an action potential under control conditions whereas it caused extensive firing when PKC-dependent ionic currents were modulated. **(C)** Omnidirectional trial of various ionic current modulation effects on rheobase (open columns) and F-I slope (solid columns). Peak currents were reduced (arrows to the right) or increased (arrows to the left) by 25 or 50%. All tested modulations failed to reproduce our *ex vivo* results (leftmost columns). **(D)** Ionic current modulations matching described PKC effects. Our *ex vivo* results were reproduced when known PKC modulated currents mediated by hyperpolarization-activated cyclic nucleotide gated channel subunit HCN1 (*I*_h_), M-type (*I*_M_), large conductance calcium-activated potassium channels BK (*I*_BK_) and persistent sodium channels Na(p) (*I*_Nap_) were altered (compare leftmost columns in (C) with (D)). In detail, the F-I slope increased to 116 Hz/nA (1.56 × initial value) and the rheobase decreased to 146 pA (0.48 × initial value). For *I*_Nap_ we modulated the V_1/2_ instead of the peak conductance (as in (C)). An orchestrated modulation of all PKC-dependent channels is necessary to reproduce the effect since trials in which we merely altered one, two or three of the four PKC-modulated currents failed to do so.

The model layer 5 pyramidal neuron was constructed using published morphological and passive membrane properties ([19], Figure 1A therein). In short, the specific intracellular resistivity R_in_ (68.0 Ω) and specific membrane capacitance (1.5 μF/cm^2^) were distributed uniformly. The membrane resistance was assigned to be sigmoidal throughout the dendrites and ranged from 36.0 kΩ cm^2^ to 5.4 kΩ cm^2^ (soma to distal dendrite tips). Spines were incorporated by decreasing membrane resistance and by increasing membrane capacitance and active channel conductances twofold, starting at a distance of 20 μm for basal dendrites and at a distance of 100 μm for the apical trunk.

To account for the specificity of the type I IFN effect to the hyperpolarization-activated cyclic nucleotide (HCN)-gated channel subunit HCN1, we substituted the original HCN channel description [19] with a specific model of HCN1 and HCN2 channels [17]. In addition, we developed a large-conductance calcium-dependent potassium (K_BK_) channel model that incorporates simultaneous dependencies of this channel on voltage and calcium concentration (see Additional file 1 and Additional file 2: Figure S1).

Descriptions of remaining channels were adopted from existing studies [20-24]. We modified channel conductance to reconstruct *ex vivo* firing behavior (all conductances in pS/μm^2^): ion channels in the simulated axon included large voltage-gated sodium conductance (Na_x_, 3500), resembling the (in-)activation properties of the sodium channels specifically found in the axon initial segment (Na_x_-type in [21]). In addition, the axon contained potassium channels of the delayed rectifier type (K_V_, 40) and of the slowly activating and non-inactivating M-type (K_M_, 50) ([20,22], respectively).

In the soma, the voltage-gated sodium conductance (Na) was set to 420 (Na-type in [21]). Furthermore, the soma included a persistent sodium channel (Na_P_, 10) component [24]. Potassium channels consisted of K_V_ (20), K_M_ (10), K_BK_ (0.6) and A-type potassium channels (K_AP_, 150) with properties that are found in the proximal region *in vitro* [23]. The high voltage activated (Ca) calcium channel conductance [23] was set to 2. The HCN conductance (0.95) consisted of two-thirds HCN1 subtype and one-third HCN2 subtype, respectively [17].

For the dendrites, Na and K_A_ channels were distributed differently in the apical and basal dendrites [23]. The Na^+^ conductance (same channel type as in the soma) decreased linearly from 150 by a factor of 0.5 in the basal dendrites, whereas it was set to 350 (proximal) and 320 (distal) in apical dendrites. All A-type potassium channel conductances started with a proximal variant (K_AP_) that was exchanged with a distal variant (K_AD_, differing by the V_1/2_ of the activation curve). This exchange occurred linearly within the first 300 μm of the dendrites [25]. The net amount of channels of both types was set to 300 for the apical dendrites. In the basal dendrites the density increased linearly from 150 with a factor of 0.7 [23]. The other channel conductances were similar for both apical and basal dendrites and the channels were of the same type as in the soma. K_V_ channels were distributed in an exponentially decreasing fashion (with a length constant of 80 μm) and a proximal starting value of 20. Only the proximal part (first 100 μm of the apical dendrite, first 20 μm of the basal) of dendrites included M-channels (5). K_BK_ and Ca^2+^ channel densities were set uniformly along all dendrites to values of 0.6 and 2, respectively. Low voltage activated calcium conductance (IT2) [23] was distributed solely on distal parts of dendrites (0.5). Net HCN conductance (two-thirds HCN1 and one-third HCN2) was distributed exponentially across compartments with a 40-times increasing density starting from 0.95 and having a length constant of 323 [17,20]. Nominal temperature was set to 32°C. Simulations were performed in NEURON (version 7.1, available at http://www.neuron.yale.edu/neuron/ [26]) and model code is available on ModelDB (http://senselab.med.yale.edu/ModelDB - accession number 168148).

## Results

### *In silico* modulation of known protein kinase C-linked channels reproduces IFN effects

Application of IFN-β increases the suprathreshold excitability of layer 5 pyramidal neurons in two ways [5]. First, the current threshold for triggering action potentials (rheobase) drops. Second, the number of action potentials elicited at fixed input current levels (gain or slope of the F-I curve) increases. To identify necessary channel modulations underlying this effect we utilized a morphological realistic model of a layer 5 neuron (Figure 1A) and adjusted its conductances to match the firing behavior observed *ex vivo* (Figure 1B top). We then separately modulated various conductances that could account for the changes in firing behavior seen upon type I IFN application. None of these separate modulations were sufficient to fully reproduce the excitability changes induced by IFN-β *ex vivo* (some examples are depicted in Figure 1C). In particular, previously shown modulation of HCN1 mediated *I*_h_ [17] alone only marginally altered firing behavior despite its role in setting resting membrane potential and input resistance. Also combined modulation of HCN channels with various other channels did not alter firing behavior in a manner comparable to our previous *ex vivo* studies.

Subsequently, we speculated that activation of PKC could link the type I IFN signaling cascade to the neuromodulatory effect. Activation of PKC has been shown to modulate various ion channels (see Introduction). We implemented these findings by reduction of: 1) HCN1 conductance to 42.5% [17]; 2) M-type potassium conductance to 76%; 3) BK calcium-dependent potassium conductance to 50%; and 4) shifted the V_1/2_ of the persistent sodium conductance by –2 mV. This combination of ion channel modulations reproduced the change in firing behavior observed upon IFN-β application *ex vivo* (Figure 1D green). Trials where we only modulated a subset of channels showed that every single channel modulation is necessary but only the combined effect of all modulation is sufficient to reproduce the entire effect (Figure 1D black). This also suggested a ranking of contribution: *I*_h_ < *I*_M_ < *I*_BK_ < *I*_Nap_.

The *in silico* results indicate that the change in firing behavior upon type I IFN application is indeed due to multiple ion channel modulations. A common characteristic of all affected ion channels is their modulation by PKC, a kinase activated by type I IFNs.

### Pharmacological protein kinase C activation corroborates *in silico* predictions for suprathreshold excitability changes in neocortical pyramidal neurons

To directly test our model-supported hypothesis that the concerted influence of PKC modulated conductances affects the excitability of cortical pyramidal neurons, we subsequently performed a number of *ex vivo* experiments in slices containing the somatosensory cortex of rats. We started with applying membrane permeable PKC activators during *ex vivo* somatic whole-cell patch-clamp recordings because if type I IFN effects are mediated by PKC, direct pharmacological PKC activation should mimic the type I IFN effects.

**Figure 2:**
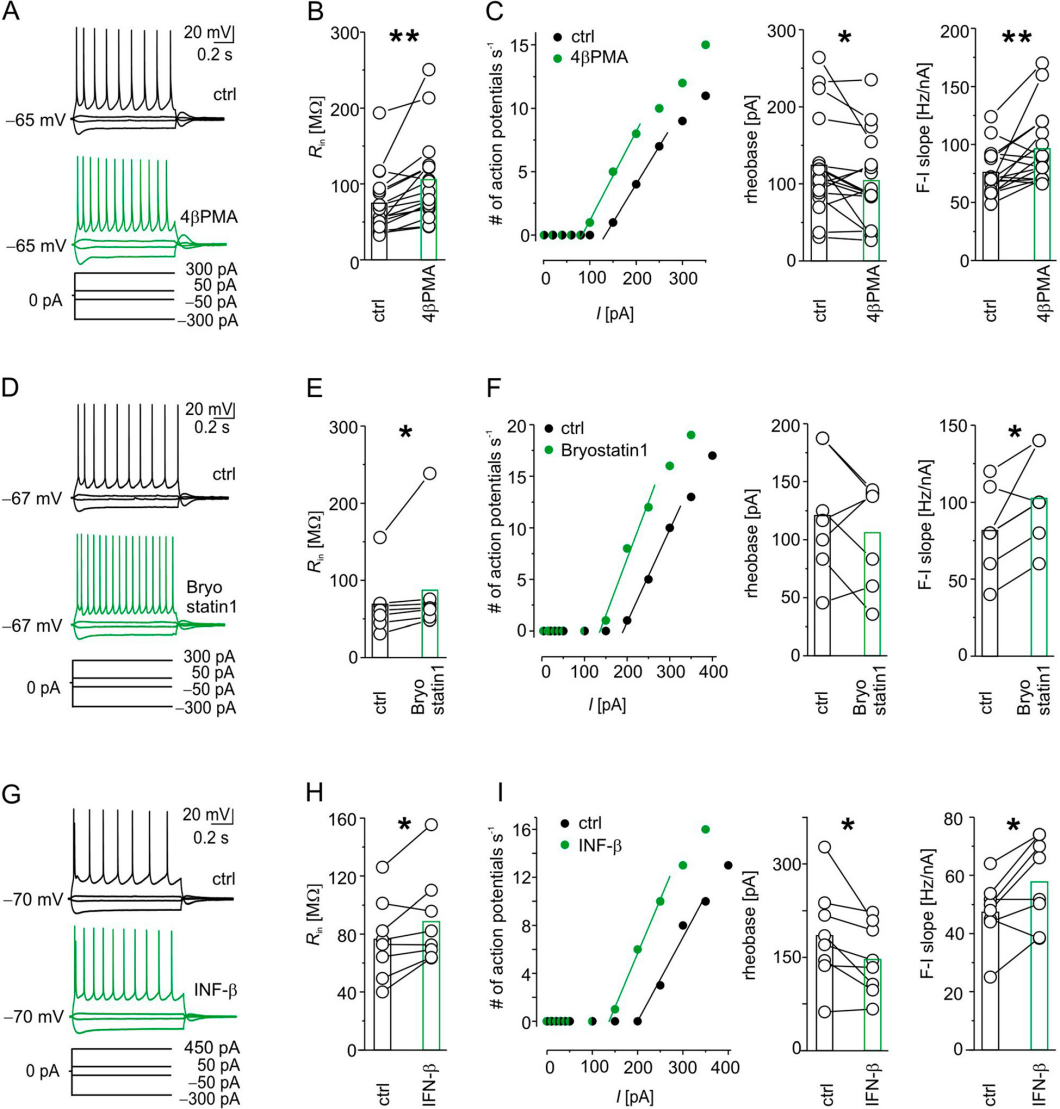
***Ex vivo*** **whole cell recordings in neocortical rat slices prove that pharmacological protein kinase C activation with 4β-phorbol 12-myristate 13-acetate or Bryostatin1 increased the suprathreshold excitability of layer 5 pyramidal neurons, thereby reproducing the effect of IFN-β. (A), (D)** and **(G)** Example trace of layer 5 pyramidal neurons in current-clamp recorded before (top) and after application of **(A)** 1 μM 4β-phorbol 12-myristate 13-acetate (4β-PMA), **(D)** 1 μM Bryostatin1 or **(G)** 1,000 IU ml^−1^ IFN-β (middle). In all *in vitro* current-clamp experiments we injected 1-second long rectangular current pulses with an increment of 50 pA and an interval of 5 seconds. For the sake of clarity, merely the voltage responses to the current injections indicated at the bottom are depicted in this and the following figures. The initial input resistance and capacitance of the depicted neurons were **(A)** 43 MΩ and 250 pF, **(D)** 45 MΩ and 230 pF and **(G)** 50 MΩ and 167 pF, respectively. **(B)**, **(E)** and **(H)** Population data demonstrate an increase of input resistance (R_in_) under respective protein kinase C (PKC)-activating conditions. **(C)**, **(F)** and **(I)** Action potential rate of a respective example neuron plotted as a function of the input current (left). Population data demonstrate a decreased rheobase for 4β-PMA and IFN-β or a tendency of decrease for Bryostatin1 **(F)** (middle) and an increase of the F-I slope (right) following application of PKC activators or IFN-β. Note that we restricted our fit to the first linear part of the F-I plot, because we regard this part as physiologically most relevant. *P < 0.05, **P < 0.01. ctrl, Control.

Indeed, applying 1 μM of 4β-PMA (Figure 2A-C), the high affinity stereo selective agonist of the cysteine rich C1/4β phorbol binding pocket [13], for 30 minutes increased the neuronal input resistance by about 40% (Table 1, Figure 2B), and the slope of the F-I curve by about 26%. The rheobase decreased by about 17% (Table 1, Figure 2C). Consistency of series resistance (R_S_, Table 1) throughout the entire recording excluded technical bias.

**Table 1:** **Influence of different protein kinase C activators and inhibitors on neocortical layer 5 neurons**

	**Control versus 4β-PMA**	**Control versus Bryostatin1**	**Control versus IFN-β**	**Control versus GF109203X + IFN-β**	**calphostin C versus calphostin C + IFN-β**
Experiments (n)	17	7	8	9	12
Input resistance (MΩ)	74.8 ± 9.8 vs 105.1 ± 13.6	69.13 ± 15.3 vs 87.3 ± 25.5	76.5 ± 9.7 vs 89.1 ± 11.1	68.1 ± 11.2 vs 73.3 ± 10	67.7 ± 10.3 vs 74.2 ± 11.9
	(*P* < 0.01^†^)	(*P* < 0.05^†^)	(*P* < 0.05^#^)	(*P* = 0.4^#^)	(P = 0.9^†^)
Rheobase (pA)	124.1 ± 16 vs 103.5 ± 14.1	120.8 ± 20 vs 105.6 ± 17	185.2 ± 28 vs 146.9 ± 20	148.3 ± 29 vs 140.6 ± 24	170 ± 19 vs 200 ± 17
	(*P* < 0.05^#^)	(*P* = 0.2^†^)	(*P* < 0.05^#^)	(*P* = 0.7^#^)	(*P* = 0.1^#^)
F-I slope (Hz/nA)	76 ± 4.9 vs 96 ± 7.5	81 ± 10 vs 103 ± 11	47.7 ± 3.9 vs 57.9 ± 5.3	91.1 ± 9.9 vs 87.8 ± 6.8	100 ± 11.8 vs 104 ± 9.6
	(*P* < 0.01^†^)	(*P* <0.05^†^)	(*P* < 0.05^#^)	(*P* = 0.7^#^)	(*P* = 0.5^#^)
After-hyperpolarization (mV)	5.8 ± 0.3 vs 2.7 ± 0.3	6.4 ± 0.64 vs 3.6 ± 0.42	5 ± 0.25 vs 3.4 ± 0.5	3.7 ± 0.44 vs 3.1 ± 0.4	3.9 ± 0.5 vs 2.6 ± 0.4
	(*P* < 0.005^#^)	(*P* <0.05^†^)	(*P* < 0.01^#^)	(*P* = 0.1^#^)	(*P* < 0.005^#^)
Series resistance (MΩ)	7.99 ± 0.27 vs 8.5 ± 0.47	8.89 ± 0.56 vs 8.94 ± 0.67	7.8 ± 0.3 vs 8.1 ± 0.4	8.5 ± 0.6 vs 8.2 ± 0.6	8.9 ± 0.5 vs 12.8 ± 2.9
	(*P* = 0.3^#^)	(*P* = 1^†^)	(*P* = 0.2^†^)	(*P* = 0.7^#^)	(*P* = 0.8^†^)

^#^Paired *t*-test; ^†^Wilcoxon signed rank test. 4β-PMA, 4β-phorbol 12-myristate 13-acetate.

To investigate whether these changes are indeed due to activation of PKC or whether they are 4β-PMA specific, we used another PKC activator, Bryostatin1 (Figure 2D-F). Bryostatin1 activates classic and novel PKCs and belongs to the group of macrocyclic lactones [27]. Comparable to 4β-PMA, Bryostatin1 (1 μM) increased the input resistance by about 26% (Table 1, Figure 2E) after 30 minutes of application. In addition, it also augmented the F-I slope by about 26% and by trend decreased the rheobase to 12% (Table 1, Figure 2F). Bryostatin1 did not significantly affect R_S_ throughout the recording period (Table 1).

In summary, two different PKC activators grossly mimicked the changes we saw previously upon IFN-β application [5] and upon *in silico* modification of PKC-dependent conductances, suggesting that PKC mediates suprathreshold IFN-β effects.

### Influence of IFN-β on suprathreshold excitability persists under whole-cell recording conditions

A necessary condition for the link between PKC activation and suprathreshold type I IFN effects is the independence of the effects on recording conditions - that is, excitability changes found with sharp microelectrodes should be reproducible under whole-cell recording conditions. Consistent with the findings obtained with sharp microelectrodes [5] and under whole-cell conditions with pharmacological PKC activation (this study), IFN-β augmented the neuronal excitability (Figure 2G-I). In detail, IFN-β increased the input resistance by about 17% (Table 1, Figure 2H) and the F-I slope by about 22% (Table 1, Figure 2I). The rheobase decreased to 21% (Table 1, Figure 2I). Also here R_S_ remained constant (Table 1).

The effect was not as prominent, although qualitatively similar, as recorded with sharp microelectrodes (R_in_: +17% versus +142%; rheobase: −22% versus −50%; F-I slope: +21% versus +60%). This could be due to an incomplete dilution of cytosolic contents [14], presumably close to the recording pipette, but also suggests that in areas remote from the recording pipette and in particular in close proximity to the cell membrane putative PKC-mediated mechanisms still work. Specifically, the anchoring of PKCs to the cell membrane by A-kinase anchoring proteins, enabling PKC-ionic current interactions (for example, for *I*_M_ [28]), supports this view. Alternatively, the effect difference between recording conditions could result from the wide age range of animals used in the present study and the consequential disparities in the level of ionic conductance development and/or distribution. However, the effect size of IFN-β on the input resistance had a tendency towards a negative correlation to age (R_in_: r = −0.66, *P* = 0.07); that is, the R_in_ of younger neurons seemed to respond slightly more to IFN-β. Nevertheless, IFN-β effects on rheobase (r = 0.52, *P* = 0.2) and F-I slope (r = −0.19, *P* = 0.6) did not depend on the age of the animals. Likewise, direct PKC activation by pharmacological activators appeared not to be correlated to the age of the animals (R_in4β-PMA_ r = −0.03, *P* = 0.89; R_inBryostatin1_ r = 0.11, *P* = 0.8; rheobase_4β-PMA_ r = 0.02, P = 0.9; rheobase_Bryostatin1_ r = 0.19, *P* = 0.7; F-I slope_4β-PMA_ r = −0.39, *P* = 0.1; F-I slope_Bryostatin1_ r = 0.27, *P* = 0.56).

Thus, modulating multiple ionic conductances by PKC exerts an age-independent effect on neuronal excitability. This is also true for the IFN effect mediated by PKC despite a slight tendency towards larger IFN-β effects on R_in_ in more juvenile animals.

### Protein kinase C activation reduced *I*_M_ and consequently decreased after-hyperpolarizations

**Figure 3:**
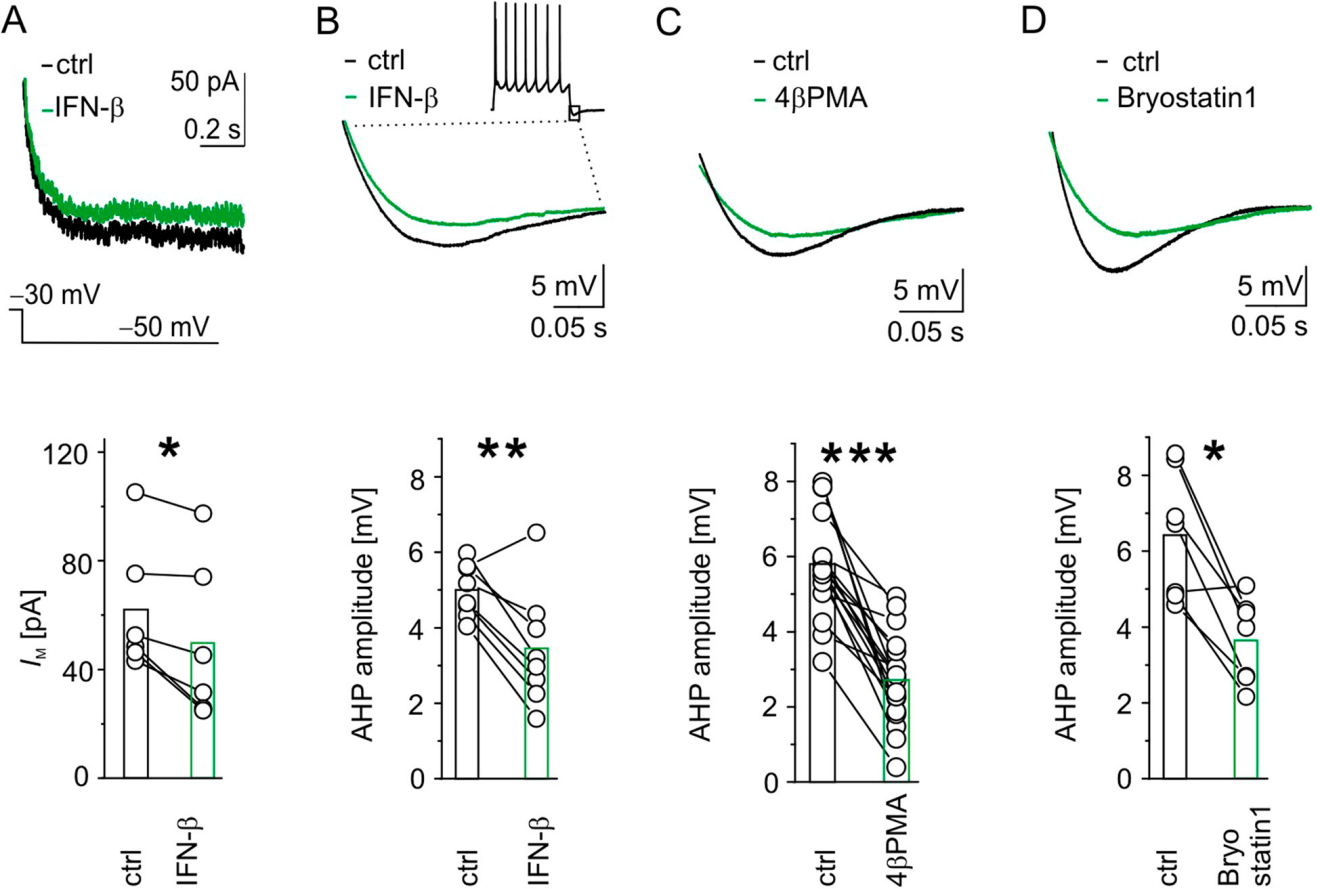
**IFN-β reduced** ***I***_**M**_ **and accordingly attenuated amplitudes of after-hyperpolarizations, as did pharmacological protein kinase C activation in layer 5 pyramidal neurons on neocortical** ***ex vivo*** **rat slices under whole-cell recording conditions. (A)** Voltage-clamp recordings of *I*_M_ tail currents depicted at the deactivating voltage of –50 mV following a 30-second depolarization to –30 mV (top) in a layer 5 pyramidal neuron before (black trace) and during (green trace) application of 1,000 IU ml^−1^ IFN-β. Population data of IFN-β-mediated reduction of *I*_M_ (bottom). **(B-D)** Current-clamp recordings reveal after-hyperpolarizations (AHP) following a train of action potentials at similar suprathreshold depolarization (top) without (black traces) and with activation of protein kinase C (PKC) by 1,000 IU ml^−1^ IFN-β **(B)**, 1 μM 4β-phorbol 12-myristate 13-acetate (4β-PMA) **(C)**, and 1 μM Bryostatin1 **(D)** (green traces). We estimated AHP amplitudes as difference between the steady state of tail current and the largest hyperpolarization of AHP. Population data show a consistent reduction under all PKC activating conditions (bottom). *P < 0.05, **P < 0.01, ***P < 0.005. ctrl, Control.

If IFN-β exerts its effects via PKC activation, PKC modulated currents should also be directly affected by IFN-β. As proof of principle we studied *I*_M_ because: 1) it determines neuronal firing behavior [29]; 2) it modulates *I*_h_ effects on neuronal integration [30]; and 3) previous pharmacological experiments suggested a modulation of this current by IFN-β [5]. To investigate *I*_M_ we blocked confounding currents, such as T-type *I*_Ca_, *I*_Na_ and *I*_h_ by 10 μM LaCl_3_, 1 μM TTX and 50 μM ZD7288, respectively, and recorded *I*_M_ tail currents before and after bath application of 1,000 IU ml^−1^ IFN-β. Under such conditions, *I*_M_ was reduced by 24% (*I*_Mctrl_ = 61.9 ± 24.1 pA versus *I*_MIFN-β_ = 49.9 ± 29.7 pA; n = 6; *P* < 0.05; Figure 3A). R_S_ did not change in these experiments (R_Sctrl_ = 6.6 ± 0.5 MΩ versus R_SIFN-β_ = 6.6 ± 0.6 MΩ; n = 6; *P* = 0.4).

One of the functional consequences of *I*_M_ is its contribution to after-hyperpolarizations (AHPs) [31]. This contribution also applies for *I*_h_, another current regulated by both type I IFNs [17] and PKC [14]. Therefore, we hypothesized that PKC activation affects AHP. Hence, we recorded AHPs after a train of action potentials elicited by injected currents between 200 and 450 pA, respectively. Comparing respective amplitudes of AHPs after similar current injections, before and after activation of PKC, yielded a clear amplitude reduction. In detail, pharmacological PKC activation by 4β-PMA decreased AHP amplitudes by about 53% (Table 1, Figure 3C). PKC activation by Bryostatin1 attenuated AHP amplitudes by 43% (Table 1, Figure 3D). Similarly, application of IFN-β reduced the AHP amplitude by about 31% (Table 1, Figure 3B).

Taken together, the IFN-β-induced reduction of *I*_M_ - a current, which is also reduced by PKC - further hints at the mediation of type I IFN effects by PKC as proposed in our model. The reduction of *I*_M_ - presumably together with the attenuation of *I*_h_ - reduces AHPs and therefore partially contributes to the observed excitability increase.

### Voltage threshold for action potential generation under pharmacological or IFN-β induced protein kinase C activation

**Figure 4:**
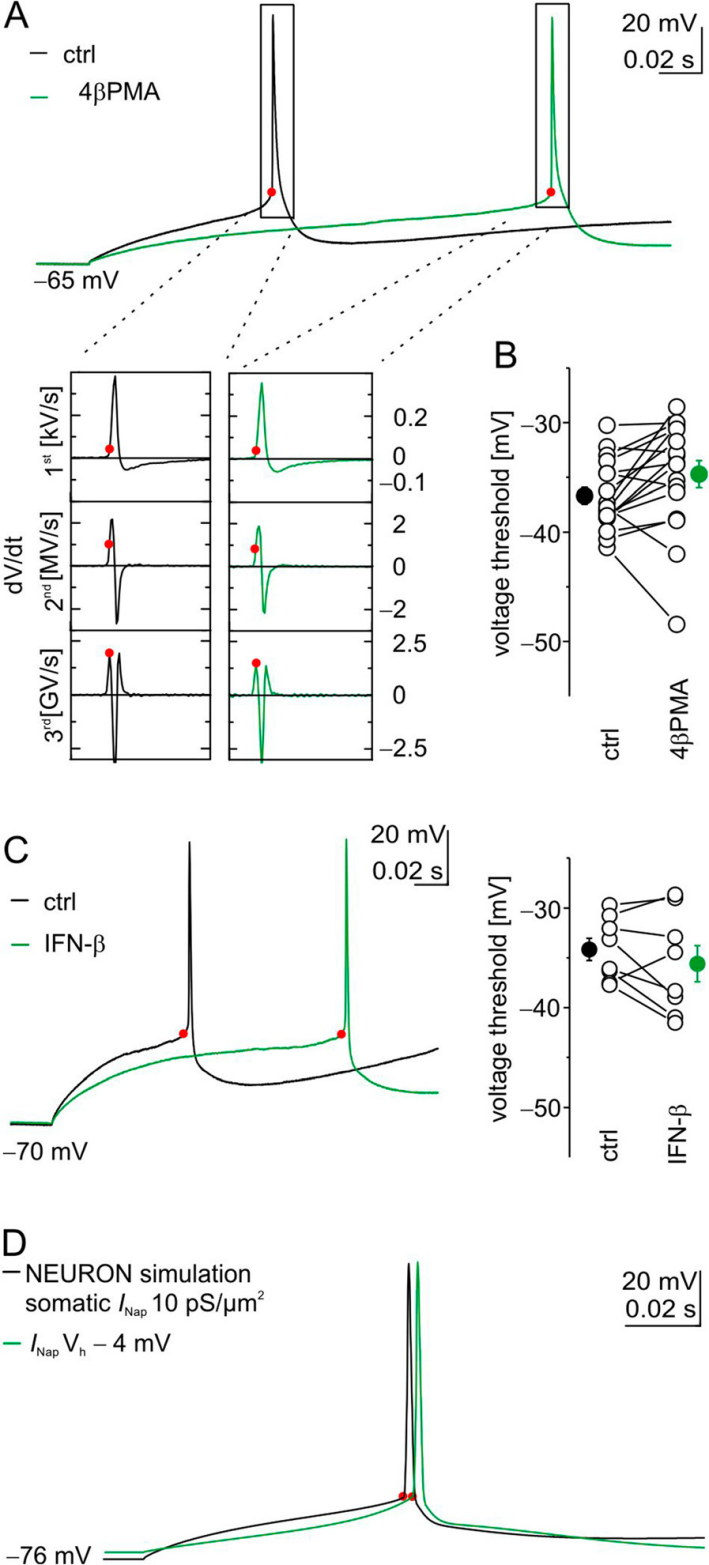
**Voltage threshold for action potential generation is unchanged by 4β-phorbol 12-myristate 13-acetate or IFN-β in** ***ex vivo*** **whole-cell recordings of layer 5 neocortical neurons and** ***in silico*** **. (A)** Voltage traces showing the first action potential with (green trace) and without (black trace) protein kinase C (PKC) activation (top). Note that due to the rheobase shift (Figure 2A) the first action potential appeared at a current injection of 150 pA under control conditions (black trace) but at 100 pA following 4β-phorbol 12-myristate 13-acetate (4β-PMA) (1 μM) application (green trace). Analysis of voltage thresholds are from the above recordings (according to [32]). The membrane potential at the time of the first peak of the third derivate of the action potential is taken as voltage threshold (bottom). Dots represent time points for estimating voltage thresholds throughout the figure. **(B)** Population data demonstrate similar voltage thresholds before and during PKC activation with 4β-PMA. **(C)** Comparable voltage thresholds for action potential generation in voltage trajectories before (black trace) and after 30 minutes of treatment with 1,000 IU ml^−1^ IFN-β (green trace, left). Again, rheobase shift caused the first action potential to appear already at 220 pA under IFN-β instead of at 350 pA as under control conditions. Population data confirm similar voltage thresholds (right). **(D)** *In silico* hyperpolarizing V_1/2_ of *I*_Nap_ (adopted from [24]) in our NEURON model by 4 mV does not alter the voltage threshold for action potential generation. ctrl, Control.

Previous studies revealed inconsistent results regarding effects of PKC activation on the voltage threshold of action potential generation. To investigate this, we estimated the voltage threshold for action potential generation in layer 5 neurons in addition to the respective current threshold (rheobase). As exemplified for 4β-PMA, we determined the voltage threshold by taking the membrane potential at the time of the first peak in the third derivate of the action potential voltage [32] (Figure 4A) and found the voltage threshold for action potential generation unchanged when we activated PKC with 4β-PMA (–37 ± 0.7 mV under control conditions versus –34 ± 1.2 mV under 1 μM 4β-PMA; *P* = 0.06, paired *t*-test; Figure 4A,B). Comparable to this and to our previous experiments with sharp microelectrodes [5], IFN-β application for 30 minutes also left the voltage threshold for action potential generation unchanged in our whole-cell experiments (–34 ± 1.1 mV under control conditions versus –35 ± 1.8 mV under 1,000 IU ml^-1^ IFN-β; n = 8; *P* = 0.2, paired *t*-test; Figure 4C). Consistent with this, the voltage threshold was left unperturbed even when we only increased *I*_Nap_ by shifting its voltage sensitivity towards hyperpolarization (Figure 4D) and under all other simulations (Additional file 3: Figure S2) in our *in silico* model. However, voltage thresholds for action potential generation in neurons treated with Bryostatin1 became slightly depolarized from –39 ± 1.4 mV under control conditions to –37 ± 1.2 mV under the influence of 1 μM Bryostatin1 (*P* < 0.05, Wilcoxon signed rank test).

Our *ex vivo* and supporting *in silico* experiments provide evidence that the concerted action of PKC-dependent intrinsic currents does not hyperpolarize the voltage threshold for action potential generation in layer 5 pyramidal neurons of the somatosensory cortex. Under certain conditions (Bryostatin1 might activate another set of PKCs) activation of PKC even slightly depolarizes the voltage threshold.

### Protein kinase C inhibition prevents IFN-β effects

**Figure 5:**
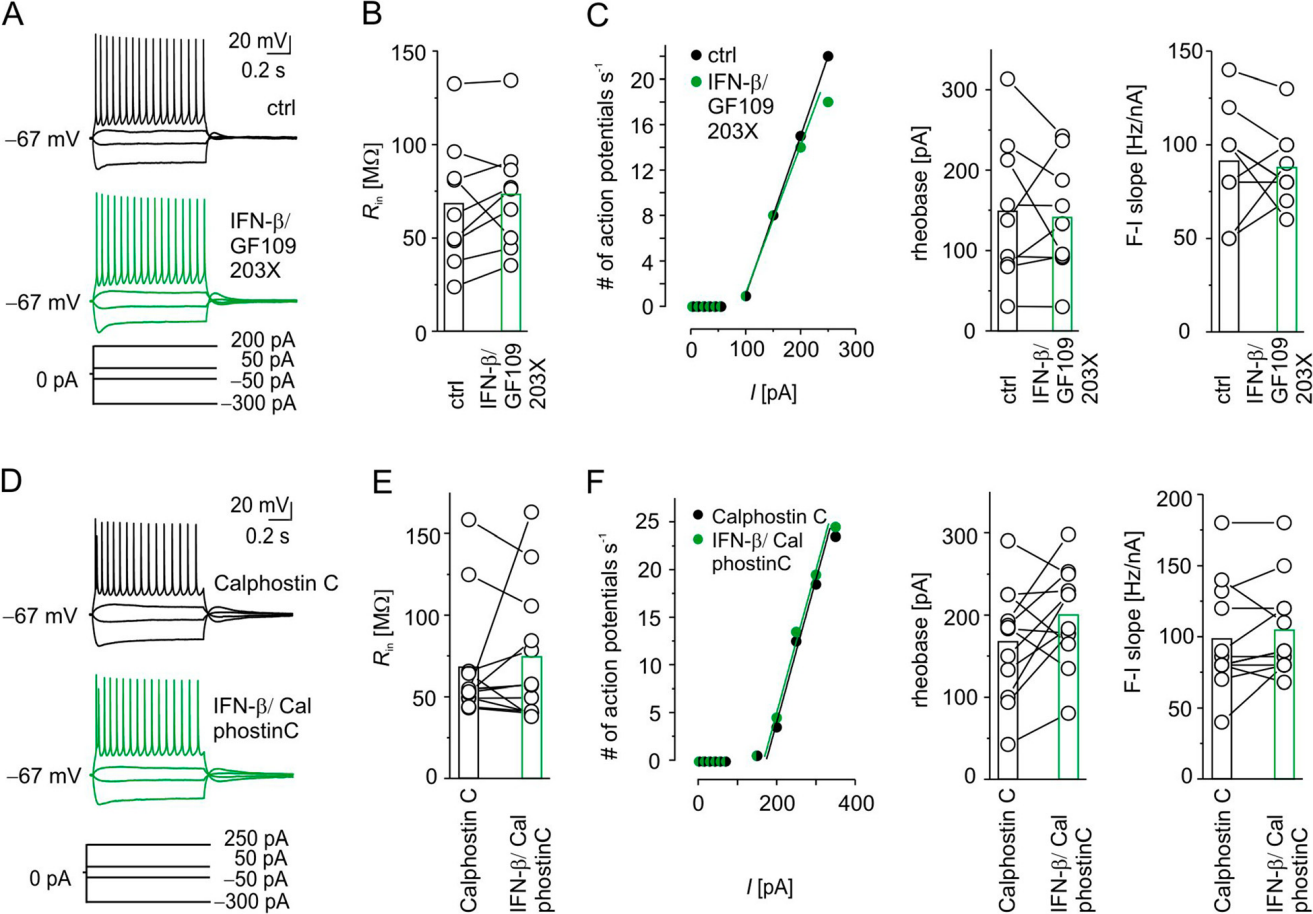
**Blocking protein kinase C activation prevents IFN-β effects on excitability of neocortical layer 5 neurons in** ***ex vivo*** **whole-cell recordings. (A)** Family of voltage traces from a layer 5 neuron recorded in current clamp mode compared with under control conditions (artificial cerebrospinal fluid, black traces, top) and following 30 minutes co-application of protein kinase C (PKC) inhibitor 1 μM GF109203X and 1,000 IU ml^−1^ IFN-β (green traces, middle) yield no obvious differences in firing behavior. The initial input resistance and capacitance of the depicted neuron were 132 MΩ and 130 pF, respectively. **(B)** Population data reveal similar input resistance (R_in_), suggesting that PKC activation is necessary for subthreshold IFN-β effects. **(C)** Rate of action potentials of a layer 5 neuron plotted against the strength of rectangular current injections (left). Population data show that rheobase (middle) and F-I slope (right) remain comparable to control values when IFN-β was co-applied with GF109203X. **(D)** Example traces of a layer 5 neuron after pre-incubation with 100 nM calphostin C (black traces) and 20 minutes after application of IFN-β (1,000 IU ml^-1^) (green traces) revealed also no difference in firing behavior. The initial input resistance and capacitance of the depicted neuron were 158 MΩ and 157 pF, respectively. **(E)** Population data of R_in_ confirmed that PKC activation is needed for IFN-β effects. **(F)** Number of action potential plotted against the strength of rectangular current injection (left). Population data indicate no difference of rheobase (middle) or F-I slope (right) between pre-incubation of calphostin C and 20 minutes of IFN-β administration. ctrl, Control.

If IFN-β modulates neuronal excitability entirely via PKC activation, blocking the latter should preclude any IFN-β effects. To test this we first co-applied the potent PKC inhibitor GF109203X (1 μM, [33]) and IFN-β (1,000 IU ml^-1^, Figure 5A-C). This treatment neither affected input resistance, nor rheobase nor F-I slope after 30 minutes. In line with this, co-application of GF109203X and IFN-β left the AHP unchanged. Again, R_S_ was similar throughout the recording (Table 1). We encountered two drawbacks in using GF109203X. First, as a broad PKC inhibitor, GF109203X disabled a longer pre-incubation due to quick deterioration of the neurons. Second, because GF109203X acts at the catalytic domain, binding to scaffold protein as AKAP might protect bound PKC from inhibition [28]. Therefore we subsequently used calphostin C, a potent, irreversible and light-dependent PKC inhibitor [34], which acts at the regulatory domain of PKC [35,36] and is insensitive to protein binding. Further, pretreatment of >1 hour did not deteriorate neuronal properties. After pre-incubation with calphostin C, IFN-β influenced neither the input resistance (Table 1, Figure 5E), nor the slope of the F-I curve, nor the rheobase (Table 1, Figure 5F). Consistency of R_S_ throughout the recording (Table 1) excluded technical bias. Since the pre-incubation period prohibited a comparative whole-cell analysis (that is, initial whole cell recordings in the absence of calphostin C) we cannot exclude intrinsic actions of calphostin C putatively preventing IFN effects independent of PKC blockade.

Moreover, in contrast to GF109203X, pretreatment with calphostin C could not prevent the IFN-β-induced reduction of post-train AHP amplitudes by about 33% (Table 1). Even if the effect of IFN on *I*_M_ might be properly blocked with calphostin C there appear to be other players controlled by PKCs not blocked by calphostin C (but putatively by GF109203X): size and shape of post-train AHPs depend on several currents such as *I*_h_, *I*_M_, *I*_KCa_ (SK-type) and other *I*_KCa_. Some of these might additionally be modulated by protein phosphorylation putatively in some cases independent of PKC [37].

However, the lack of major IFN-β effects on neuronal excitability when PKC activation was blocked adds further evidence for PKC mediation of type I IFN actions.

## Discussion

We performed this study in search of a mechanism linking IFN-β to its boosting effects on neuronal excitability. Resulting data provide a line of evidence for PKC activation as a unitary mechanism for type I IFN-mediated modulation of excitability in layer 5 pyramidal neocortical neurons. (1) An *in silico* model based on our previous blocking experiments [5] endorsed our hypothesis that only a concerted modulation of HCN1-mediated *I*_h_, *I*_M_, *I*_BK_ and *I*_Nap_ reproduce all type I IFN effects on neuronal excitability. All these channels are also modulated by activated PKC. (2) *Ex vivo* pharmacological activation of PKC in somatosensory layer 5 pyramidal neurons by 4β-PMA or Bryostatin1 increased the neuronal excitability similar to IFN-β application under whole-cell recording conditions. (3) We showed a reduction of post-train AHPs and confirmed a modulation of *I*_M_ by IFN-β *ex vivo*. (4) Blocking PKC activation at the catalytic or at the regulatory side prevents almost all IFN-β effects on neuronal excitability. In summary, we conclude that PKC is both sufficient and necessary for the downstream mediation of neuronal IFN-β effects.

PKC activation, a ubiquitous component in the interferon signaling cascade [2,3], had been shown to modulate all channels proposed *in silico*: it reduces *I*_M_ [12], *I*_BK_ [15] and *I*_h_ [13,14]. It also shifts the activation curve of *I*_Nap_ closer to the resting membrane potential, therefore decreasing the rheobase [16]. In our initial study [5], we discussed PKC activation as an unlikely candidate for mediating the type I IFN effects because voltage thresholds for action potential generation were unchanged, as opposed to earlier experiments following PKC activation in cortical neurons [38]. However, although PKC-dependent sodium channel phosphorylation is known to contribute to the functional balance between excitation and inhibition [39], the actual effect of PKC modulation appears rather complex and may vary in different neuronal populations and further depend on slight differences of PKC activating substances and/or co-activators on various PKC isoenzymes. Phosphorylation - whether PKC-dependent or not - variably affects *I*_Nap_ amplitude, but consistently induces a negative shift in the activation curve of *I*_Nap_ [16,24,38,40]. Although this certainly enhances its depolarizing contribution to neuronal discharges, the exact contribution to voltage thresholds remains controversial. Indeed, in layer 5 neurons, the voltage threshold for action potential generation threshold may shift much less than previously thought (about –1.5 mV [40]). In particular this might be due to concomitant PKC effects on transient sodium currents (*I*_Nat_) [40], which in turn directly influence voltage thresholds. Conflicting results have also been reported regarding the influence of PKC activation on *I*_TCa_ [41,42]. However, changes in firing behavior do not rely on a modulation of *I*_TCa_*in silico* in pyramidal layer 5 neurons.

Our study links the numerous individual studies on PKC effects on single conductances, therefore contributing to the increasingly complex picture of neuronal kinase actions. However, our conductance-based *in-silico* model was designed for gross screening and does not explicitly include all ion channels known to be present in neocortical layer 5 neurons. For example, various two-pore domain potassium conductances have been combined into one leak conductance. In addition, we neglected potassium currents through two trans-membrane domains (*I*_Kir_), small calcium-dependent potassium currents (*I*_SK_) and synaptic conductances. Moreover, the model does not account for any context-dependent roles of ionic conductances as, for instance, somato-dendritic versus axonal *I*_M_ [43]. Because our model was not intended to be exhaustive, we cannot exclude additional minor changes of conductances not incorporated in the model, particularly synaptic inputs.

In line with the time course of ion channel modulation, application of type I IFNs leads to PKC activation within 5 minutes (as shown for IFN-β [44]). Cortical neurons contain all major isoenzyme groups, such as conventional, novel, and atypical [45], but composition may differ in individual neurons. Beyond that, the PKC activators used here, 4β-PMA and Bryostatin1, might slightly differ in their action, although both bind to a C1 domain [46]. This might be responsible for slight differences in effects on cortical layer 5 neurons. However, both PKC activators act on the conventional and novel PKCs [27], and the PKC inhibitor GF109203X inhibits all three classes of PKC isoenzymes, but is more efficient on conventional and novel PKCs [46]. Therefore, we assume that the PKC isoenzymes mediating neuronal type I IFN effects belong to the conventional and/or novel class.

Inducible cytokines as, for instance, IFNs are key players in innate and adaptive immune responses that are produced in response to peripheral somatic or central nervous system inflammation [1] and are important in mood disorders [47]. The signaling pathway of IFN actions within the central nervous system is, however, poorly understood and the exact extracellular IFN protein levels during inflammation are still unknown. However, several hints suggest that cytokines generally act together in inflammation and are locally produced [1]. Therefore IFNs putatively activate their receptors even at low tissue concentrations. Neuromodulatory effects of cytokines have been discussed in regard to modulation of hormones and in regard to release and detection of neurotransmitters [47-50]. Our study shows that type I IFNs additionally modulate several neuronal ion channels, profoundly increasing neuronal excitability. In fact, our data provide an impressive confirmation of the findings that small alterations in a sensitive set of conductances have powerful effects [6] and they extend them to the mammalian central nervous system and to a putatively clinically relevant context. They also position type I IFNs in the row of neuromodulators that modify existing conductances along sensitive directions [6] via putatively membrane-bound PKCs. During central nervous system inflammations, IFN-β is produced by neurons [1] and is upregulated in myeloid cells [51]. Moreover, type I IFNs elicit responses in all cell types of the central nervous system, including neurons [1,52,53]. PKCs - at least Ca^2+^-dependent isoenzymes - are also activated by neuronal activity itself, for instance by high frequency stimulation in rat hippocampus [54]. Together this implies a kind of feed-forward mechanism, eventually leading to over-excitatory states. Extrapolating these basic functional changes to human inflammatory states might help to explain sickness behavior and increased susceptibility to depression [47,55].

Even in uninfected or non-inflamed brains, type I IFNs might play a neuromodulatory role given the basal IFN-β levels under those conditions [51].

Our data suggest that activation of PKC links the type I IFN signaling cascade to multiple neuronal ion channels. Based on this study, type I IFN can be linked to various other PKC-mediated neuronal effects such as impeding the regeneration of damaged axons [56] or even controlling dendrite arborization during development [57].

## Additional files

Additional file 1:
**Large-conductance calcium-dependent potassium (BK) channel model.**
Additional file 2: Figure S1. Steady state opening probability of simulated BK-channels. Incorporating the values from Table 1 yielded a Ca^2+^ and voltage dependence of the opening probability as depicted. The Ca^2+^ concentration is scaled to incorporate the coupling between BK and Ca^2+^ channels. Additional file 3: Figure S2. Various simulated changes in *I*_Nap_ properties (adapted from [6] to our NEURON-model); that is, V_1/2_ 2 mV hyperpolarized (grey), *I*_Nap_ conductance increased to 110% (blue), 125% (green) and 150% (red) did not change the voltage threshold for action potential generation.

## Abbreviations

4β-PMA: 4β-phorbol 12-myristate 13-acetate
ACSF: artificial cerebrospinal fluid
AHP: after-hyperpolarization
HCN: hyperpolarization-activated cyclic nucleotide gated
IFN: interferon
*I*_BK_: large conductance calcium-dependent potassium current
*I*_h_: hyperpolarization-activated cyclic nucleotide gated current
*I*_M_: M-type potassium current
*I*_Nap_: persistent sodium current
P: postnatal day
PKC: protein kinase C
R_in_: input resistance
R_S_: series resistance
sACSF: sucrose artificial cerebrospinal fluid
