# Supplementary material for: Protein kinase C activation mediates interferon-β-induced neuronal excitability changes in neocortical pyramidal neurons
Source: J Neuroinflammation. 2014 Oct 29;11:185. doi: 10.1186/s12974-014-0185-4 (PMC4222407; doi:10.1186/s12974-014-0185-4)
Supplement: Additional file 1: — Large-conductance calcium-dependent potassium (BK) channel model. [file 12974_2014_185_MOESM1_ESM.docx]

**Additional file 1**

**Large-conductance calcium-dependent potassium (BK) channel model**

Large-conductance Ca^2+^-dependent potassium channel (BK)-mediated currents were described with:

*
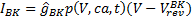

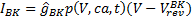
*

For given calcium concentration the current obeyed a first order kinetic:


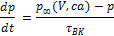


with

*
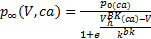

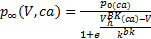
*

Varying [Ca^2+^]_i_ affected steady state open probability by: (1) increasing maximum steady state open probability [1] and (2) shifting the voltage of half maximal activation (V_1/2_) [1, 2]. That was incorporated by:

*
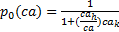

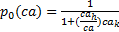
*

and


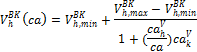


Coupling of BK^+^ and Ca^2+^ conductances leading to locally increased [Ca^2+^]_i_ [1, 3] was modeled by:


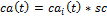


Changes of Ca^2+^-dependent parameters due to altered [Ca^2+^]_i_ took place instantaneously. Due to lack of quantitative data on channel opening kinetics and its dependence on [Ca^2+^]_i_ for BK channels time constants for channel kinetics depending on [Ca^2+^]_i_ and/or voltage were not incorporated. Instead, time constant was set to 1 ms allowing the opening probability to follow the sudden increase in [Ca^2+^]_i_ and voltage as evoked by an action potential [2].

The model was parameterized based on published values [1, 2, 4, 5]:

| 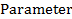 | 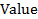 |
| --- | --- |
| 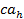 | 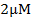 |
| 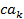 | 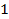 |
| 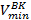 | 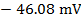 |
| 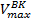 | 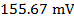 |
| 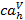 | 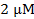 |
| 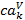 | 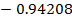 |
| 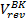 | 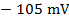 |
| 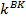 | 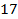 |
| 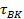 | 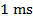 |
| 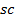 | 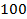 |

Table 1

**References**

1. Benhassine N, Berger T: **Homogeneous distribution of large-conductance calcium-dependent potassium channels on soma and apical dendrite of rat neocortical layer 5 pyramidal neurons.** *EurJNeurosci* 2005, **21:**914-926.

2. Womack M, Khodakhah K: **Active contribution of dendrites to the tonic and trimodal patterns of activity in cerebellar Purkinje neurons.** *J Neurosci* 2002, **22:**10603-10612.

3. Neher E: **Vesicle pools and Ca2+ microdomains: new tools for understanding their roles in neurotransmitter release.** *Neuron* 1998, **20:**389-399.

4. Gong LW, Gao TM, Huang H, Tong Z: **Properties of large conductance calcium-activated potassium channels in pyramidal neurons from the hippocampal CA1 region of adult rats.** *The Japanese journal of physiology* 2001, **51:**725-731.

5. Benhassine N, Berger T: **Large-conductance calcium-dependent potassium channels prevent dendritic excitability in neocortical pyramidal neurons.** *Pflugers Arch* 2009, **457:**1133-1145.

6. Uebachs M, Opitz T, Royeck M, Dickhof G, Horstmann MT, Isom LL, Beck H: **Efficacy loss of the anticonvulsant carbamazepine in mice lacking sodium channel beta subunits via paradoxical effects on persistent sodium currents.** *J Neurosci* 2010, **30:**8489-8501.
