# Supplementary figures and images for: Protein kinase C activation mediates interferon-β-induced neuronal excitability changes in neocortical pyramidal neurons
Source: J Neuroinflammation. 2014 Oct 29;11:185. doi: 10.1186/s12974-014-0185-4 (PMC4222407; doi:10.1186/s12974-014-0185-4)

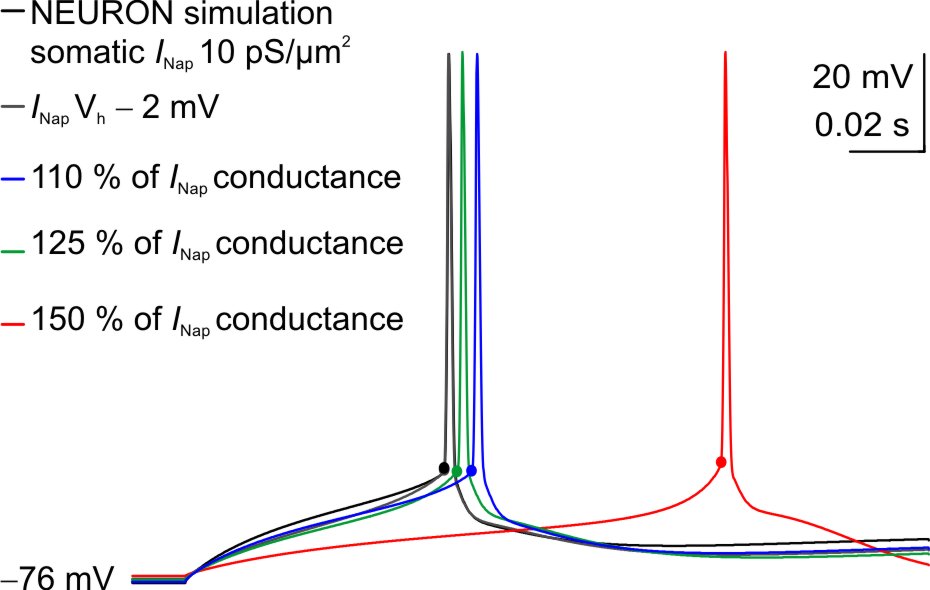

Supplement: Additional file 2: Figure S1. — Steady state opening probability of simulated BK-channels. Incorporating the values from Table 1 yielded a Ca2+ and voltage dependence of the opening probability as depicted. The Ca2+ concentration is scaled to incorporate the coupling between BK and Ca2+ channels. [file 12974_2014_185_MOESM2_ESM.jpeg]

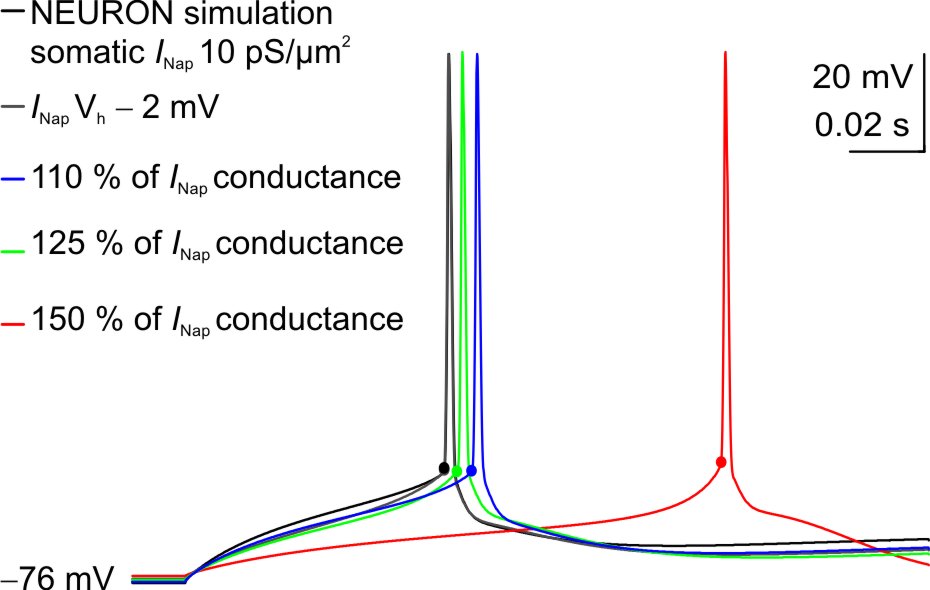

Supplement: Additional file 3: Figure S2. — Various simulated changes in I Nap properties (adapted from [6] to our NEURON-model); that is, V1/2 2 mV hyperpolarized (grey), I Nap conductance increased to 110% (blue), 125% (green) and 150% (red) did not change the voltage threshold for action potential generation. [file 12974_2014_185_MOESM3_ESM.jpeg]
